# Developmental Aspects of Cardiac Adaptation to Increased Workload

**DOI:** 10.3390/jcdd10050205

**Published:** 2023-05-10

**Authors:** Bohuslav Ostadal, Frantisek Kolar, Ivana Ostadalova, David Sedmera, Veronika Olejnickova, Marketa Hlavackova, Petra Alanova

**Affiliations:** 1 Institute of Physiology of the Czech Academy of Sciences, 142 20 Prague, Czech Republic; kolar@biomed.cas.cz (F.K.); iostadal@biomed.cas.cz (I.O.); david.sedmera@lf1.cuni.cz (D.S.); veronika.olejnickova@fgu.cas.cz (V.O.); marketa.hlavackova@fgu.cas.cz (M.H.); petra.alanova@fgu.cas.cz (P.A.); 2 Institute of Anatomy, First Faculty of Medicine, Charles University, 128 00 Prague, Czech Republic

**Keywords:** cardiac development, adaptation to overload, adaptive growth response, phylogeny, postnatal ontogeny, hypertrophy, hyperplasia

## Abstract

The heart is capable of extensive adaptive growth in response to the demands of the body. When the heart is confronted with an increased workload over a prolonged period, it tends to cope with the situation by increasing its muscle mass. The adaptive growth response of the cardiac muscle changes significantly during phylogenetic and ontogenetic development. Cold-blooded animals maintain the ability for cardiomyocyte proliferation even in adults. On the other hand, the extent of proliferation during ontogenetic development in warm-blooded species shows significant temporal limitations: whereas fetal and neonatal cardiac myocytes express proliferative potential (hyperplasia), after birth proliferation declines and the heart grows almost exclusively by hypertrophy. It is, therefore, understandable that the regulation of the cardiac growth response to the increased workload also differs significantly during development. The pressure overload (aortic constriction) induced in animals before the switch from hyperplastic to hypertrophic growth leads to a specific type of left ventricular hypertrophy which, in contrast with the same stimulus applied in adulthood, is characterized by hyperplasia of cardiomyocytes, capillary angiogenesis and biogenesis of collagenous structures, proportional to the growth of myocytes. These studies suggest that timing may be of crucial importance in neonatal cardiac interventions in humans: early definitive repairs of selected congenital heart disease may be more beneficial for the long-term results of surgical treatment.

## 1. Introduction

Adaptation to the environment is a basic attribute of all forms of life. According to the classic definition by Adolph [1], “…adaptations are modifications of organisms that occur under certain circumstances…and are not limited, as is often the case, to modifications that appear to be favorable to the individual”; otherwise, each adaptation has positive and negative consequences. If the heart is exposed to an increased workload for a long period, it usually increases its muscle mass. The basic parameter determining the functional capacity of the cardiac muscle is, therefore, the heart weight.

Cardiac adaptation to an increased workload is usually associated with dramatic changes in the circulating levels of various hormones and growth factors, as well as with remodeling of the extracellular matrix and subcellular organelles, including the sarcolemma, sarcoplasmic reticulum, mitochondria and myofibrils. A critical role in the induction of cardiac subcellular remodeling is played by changes in gene expression and the activation of proteases. Changes in the function of subcellular organelles may serve as compensatory mechanisms for the adaptation of cardiac cells [2,3].

The adaptive cardiac growth response changes significantly during phylogenetic and ontogenetic development. In poikilotherms, the cardiomyocytes proliferate during the whole life; this proliferative potential is often associated with the lifelong capability of these species to grow [4]. On the other hand, the extent of cardiomyocyte proliferation in homeotherms depends on the developmental period: while fetal or neonatal cardiac myocytes have proliferative potential (hyperplasia), proliferation declines sharply after birth, and the heart grows almost exclusively via hypertrophy of the cardiomyocytes. It is, therefore, understandable that the regulation of cardiac growth responses to the increased workload (hyperplastic or hypertrophic) thus varies markedly during ontogenetic development [5].

Interest in the developmental aspects of cardiac adaptation to increased workload still increases. The main reason is likely the effort to clarify mechanisms responsible for the plasticity of the immature heart and to discover new strategies, leading to the induction of the proliferation of adult cardiomyocytes as well as to the regeneration of the damaged myocardium after infarction [6]. Whereas a large amount of information currently exists on cardiac adaptation to overload in adults, much less is known about developmental differences in cardiac adaptive response. In this review, we summarize, therefore, some data addressing phylogenetic and ontogenetic aspects of cardiac adaptation to increased workload. Particular attention was paid to the regulation of normal cardiac growth and differences in the adaptive response of the immature and adult myocardium.

## 2. Phylogenetic Remarks

**Figure 1:**
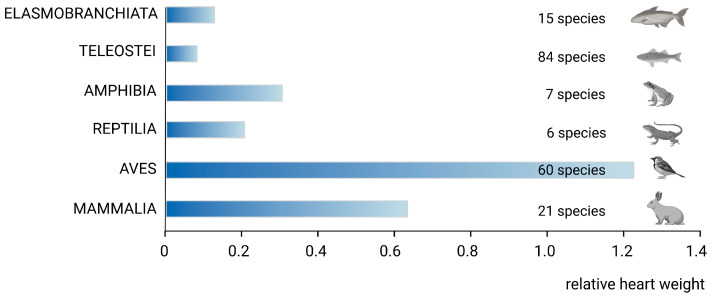
Mean values of the relative heart weight in individual classes of vertebrates. The number of species from which the mean value was calculated is given in each column (data from [7]).

The transition from water to land and the need for thermoregulation and physical activity necessary for species survival represent major adaptive changes in cardiac function during phylogeny. Heart size in different vertebrate species varies considerably. The average values of the relative heart weight (heart weight/body weight × 100) for each vertebrate class are shown in Figure 1. Relative heart weight is highest in birds, followed by mammals and cold-blooded animals. The maximal acceleration of heart growth occurs when the metabolic activity increases significantly; for example, during the transition from poikilothermy to homeothermy [2,7,8]. Another important factor represents the increased energy demand induced by antigravity workload, especially in birds.

**Figure 2:**
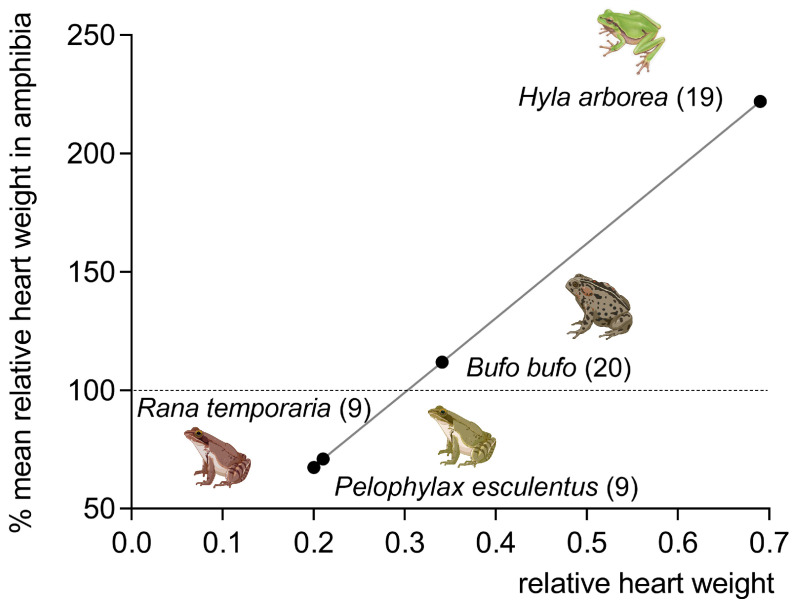
Relative heart weight in amphibia (frogs) of various mode of life; 100% means average relative heart weight in respective class. Number of animals in brackets (data from [7]).

Significant differences in relative heart weight also exist within individual classes of vertebrates: physically active poikilotherms and homeotherms have a greater relative heart weight than inactive animals [9,10]. For instance, the most active species of fish also have the largest relative heart weight. Similar differences in relative heart weights also exist in amphibians: perennial aquatic frogs *(Rana esculenta*) have a lower relative heart weight than predominantly terrestrial frogs (*Bufo vulgaris*, *Hyla arborea*). The relative heart weight of the climbing tree frog (*Hyla arborea*) even reaches values typical for mammals (Figure 2).

The relationship between heart weight and physical activity in homeotherms was studied by Clark, who also introduced the terms “athletic” and “non-athletic” animals [9]. Two species are typical examples of the important role of excessive antigravity work: the flying mammal, i.e., the bat (*Myotis myotis*), and the squirrel (*Sciurus*). They have a significantly higher relative heart weight compared to mammals of approximately the same body weight. Differences in physical activity may also explain variations in relative heart weights between domesticated and wild species [8,11]. In this context, it is important to emphasize that in all individuals increased heart weight is not a pathological hypertrophy, but a genetic adaptation to physical activity by these species. Interestingly, left ventricular pressure in newborn mammals is similar to mean ventral aortic blood pressure (30–50 mm Hg) in trout, cod and several other fish species [12]. Although fish are very different from mammals and function at lower and fluctuating body temperatures, it is interesting that the cardiomyocyte morphology, excitation–contraction (e–c) coupling and energetics are similar to those of neonatal mammals. This is, of course, a simplistic view, because the hearts of many fish species generate much lower pressures, whereas the hearts of very active tuna can generate a mean aortic pressure of up to 90 mm Hg [13]. Presumably, the ventricular wall stress has a greater influence on ventricular structure than absolute pressure. The inner spongious musculature is divided into several smaller compartments (sinusoids), characterized by lower wall stress than the large central lumen in mammalian hearts [14]. This is the reason why some authors compare neonatal mammalian cardiomyocytes with trout cardiomyocytes, as this is one of the most commonly studied fish species.

**Figure 3:**
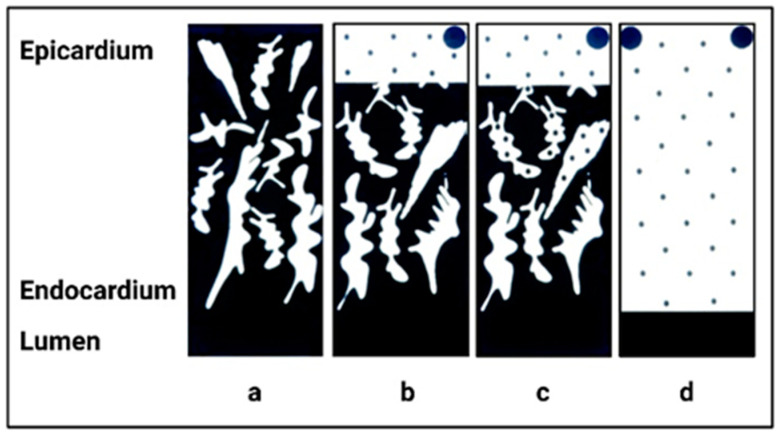
Scheme of the different types of myocardial blood supply during phylogenetic development: (**a**) spongious trabeculated musculature, entirely supplied from the ventricular cavity; (**b**) the inner avascular spongious layer is covered by an outer compact musculature with vascular supply; (**c**) as (**b**), but capillaries are also present in some trabeculae of spongious musculature; (**d**) compact musculature supplied from coronary arteries. Type (**a**–**c**)—the heart of poikilotherms; type (**d**)—the heart of adult homeotherms (adapted with permission from [21]).

**Figure 4:**
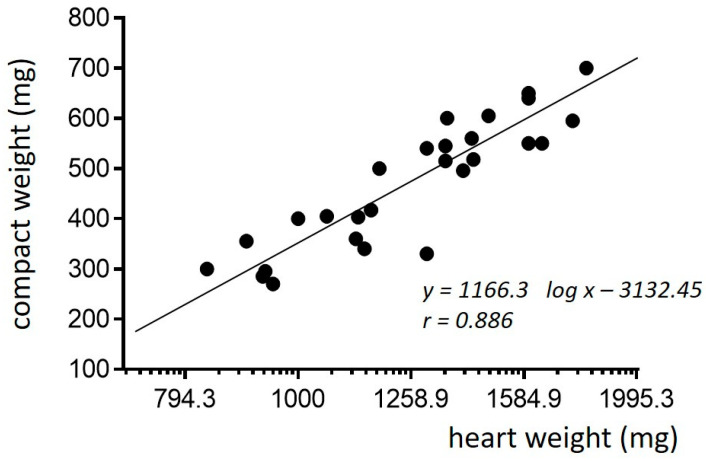
Relationship between the weight of the total cardiac musculature and its compact layer in the carp. Heart weight on the logarithmic scale (adapted with permission from [18]).

Phylogenetic differences in cardiac weight, performance, and energy metabolism are closely related to the form of oxygen pathway from the blood to mitochondria [15]. The heart of poikilotherms is either entirely spongious, supplied by diffusion from the ventricular cavity, or the inner avascular layer is covered by an outer compact myocardium supplied by capillaries from the coronary arteries (Figure 3). The thickness of the compact musculature in cold-blooded vertebrates increases with increasing heart and body weight. These findings suggest that compact musculature is necessary to maintain blood pressure in larger hearts (Figure 4, application of the law of Laplace) [2,16,17,18]. Structural differences between the spongy and compact myocardium are accompanied by significant changes in energy metabolism: the spongy myocardium is better equipped for aerobic metabolism than the compact layer [18,19]. Cardiac adaptations to different types of increased workload vary among lower vertebrates according to the structural, functional and metabolic properties of their myocardium. This can range from the isolated increase in individual layers to the enlargement of the entire heart through a combination of hyperplasia and hypertrophy of cardiomyocytes [2,20].

As mentioned above, in cold-blooded vertebrates the proliferative potential of cardiomyocytes persists throughout their lifetime, allowing the complete regeneration of the damaged heart, as has been demonstrated, for example, in urodele amphibians [22] and zebrafish [23]. An experimental model of cardiac ventricular amputation allows the identification of critical genes and pathways. Thus, understanding the factors that regulate myocyte re-entry into the cell cycle in poikilothermic hearts could help in unraveling the regenerative potential of the myocardium in humans [24].

In conclusion, a question arises as why it is useful to analyze the mechanisms of cardiac adaptation in lower vertebrates. Although one cannot entirely agree with the view that ontogenetic development is a recapitulation of phylogeny, comparative studies (more correctly labelled than “phylogenetic” because the investigator is not comparing the entire evolutionary series, but only certain classes—i.e., a *pars pro toto* approach) have contributed significantly to our understanding of the function of the cardiovascular system [11,25]. Perhaps the study of the cardiovascular system in lower vertebrates continually challenges the perceived notion that a simple function must arise from a simple structure [26].

## 3. Normal Cardiac Growth during Postnatal Ontogeny

Consistent with a developmental approach to cardiac adaptation, normal cardiac growth represents an adaptive response to increased energy demands during ontogeny. In most mammalian species, cardiac performance in terms of stroke work is lower at birth than in adults. During development, blood pressure and cardiac output increase, and the heart must reach a higher performance. Accordingly, the cardiomyocytes change their morphology, e–c coupling and energetics [27]. Achieving final organ size requires the precise coordination of cell growth, proliferation and survival throughout postnatal life [3].

**Figure 5:**
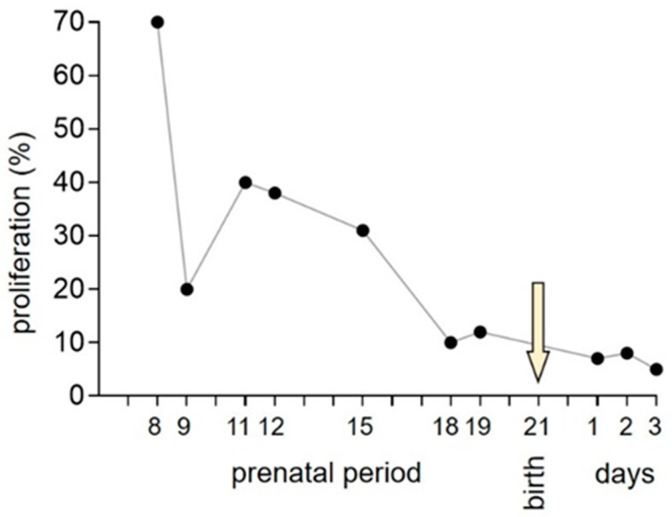
Myocyte proliferation in the developing rat heart (data from [28], adapted with permission from [5]). Arrow indicates the birth.

In mammals, normal cardiac growth is biphasic: during early development, hyperplasia of cardiomyocytes predominates, while in later life total cell mass is achieved through hypertrophic growth. The proliferation of cardiac myocytes peaks during embryonic life and then declines until birth [5,28] (Figure 5). A rapid transition from myocyte hyperplasia to hypertrophy occurs between postnatal days 3 and 4 in rat and mouse hearts [29,30]. In humans, the final cardiomyocyte number is reached as early as one month after birth [31], although other studies have reported an increase in the number of cardiomyocytes at even later stages of development [32]. Proliferative activity in the heart not only increases its mass to match the increasing demands, but along with programmed cell death and migration is a major factor in shaping the developing heart [5]. Ventricular shape is determined by the ballooning of the left and right ventricle due to a high proliferative activity in their apical parts and relative quiescence in the septum [33]. Immigrant cell populations are also important for septation division of the originally single outflow tract into the aortic and pulmonary channels by the neural crest cells. The remodeling of the atrioventricular junction is induced by the epicardially derived cells forming a good proportion of cardiac fibroblasts and contributing to atrioventricular valves [34]. Prenatal and early postnatal ventricular myocytes show generally very low levels of apoptosis. However, early postnatal rodent right ventricles have shown a temporary increase in apoptosis, interpreted as an adaptation to postnatal pressure unloading and higher oxygen tension [35]. A similar wave was described in both ventricles in lambs prior to birth using biochemical methods [36]; this preceded the switch from hyperplastic to hypertrophic growth and was shown to reduce the number of myocytes by almost 30%. The significance of this finding is unclear, and it should be corroborated by tissue-specific markers such as TUNEL to pinpoint the spatial distribution of dying myocytes. Similarly, the cause of interspecies differences is unknown, but different degrees of maturation at birth may play a role.

The adult heart is traditionally considered as a postmitotic organ, but numerous studies have demonstrated that adult mammalian cardiomyocytes have very limited proliferative potential [4,31,37,38]. Many studies have focused on promoting cardiomyocyte proliferation via inducing cell cycle re-entry, which is essential for cardiac recovery after myocardial infarction (for Ref. see [6]). Recently, Nakada et al. investigated the effect of severe chronic hypoxia on cardiomyocyte proliferation [39]. Their study was based on the presumption that during embryonic development, the mammalian heart primarily utilizes anaerobic metabolism to produce energy, and fetal cardiomyocytes have a high proliferative capacity. The transition to higher oxygen levels is associated with an interruption of the cell cycle of cardiomyocytes and a reduction in their proliferative potential. The authors have observed that a gradual reduction in ambient oxygen levels to 7% induced in adult mice a proliferative response of cardiac myocytes. Thus, hypoxemia reduced the mitochondrial oxidative metabolism and reactive oxygen species (ROS) production, repressed oxidative DNA damage, and led to cardiac hyperplasia through increased mitosis. Interestingly, high altitude populations have reduced mortality due to ischemic heart disease [40]. However, the recent study failed to reproduce the principal finding: the total number of cardiomyocytes did not differ between control and hypoxia. Moreover, indices of cardiomyocyte DNA synthesis, cell-cycle activity and cytokines failed to show a hypoxia-induced stimulation of cardiomyocytes proliferation in left ventricles [41].

Numerous studies using different methodological approaches have systematically mapped the ontogenetic development of cardiomyocyte proliferation [5,42,43]; their enumeration and critical evaluation is beyond the scope of this review. Too often, however, insufficient evidence or improper controls are provided to support the claim that cardiomyocytes proliferate, a process that should be strictly defined as the formation of two de novo functional cardiomyocytes from a single original cell. A complementary approach to the analysis of cell-cycle markers is to infer complete cardiomyocyte division by estimating the associated increase in the total number of cardiomyocytes in the entire heart, also known as “endowment”. Currently, two approaches prevail: (i) enzymatic disaggregation and quantification, and (ii) design-based stereology [43]. Due to the significantly different results obtained by enzymatic dissociation compared to in situ stereology, it is difficult to determine with certainty which method generates more precise results [43]. It should be emphasized that cardiomyocyte cell-cycle activity does not necessarily equate to proliferation, as it may also reflect hypertrophy, polyploidization, or polynucleation [44]. It should be noted that further polynucleation, usually including a high number of nuclei (four, eight or even more), can be a sign of cardiac disease/damage in older individuals. Therefore, it is important to be cautious when interpreting data from cell-cycle assays. A main problem, particularly in vivo, is to determine whether the cell-cycle marker signal actually belongs to the cardiomyocyte or to other cardiac cells. This is particularly important because there is often increased fibroblast proliferation and infiltration of the inflammatory cells. Failure to recognize this issue often leads to a misinterpretation of results and is often a cause of controversy in the fields of cardiac regeneration [42].

The postnatal growth of the normal mammalian heart depends almost entirely on cellular hypertrophy. The reduction in proliferation after birth is followed by brief bursts of other possible cell-cycle variations, known as polyploidization and binucleation. Both of these variations occur in mouse and human cardiomyocytes after birth, but in relatively different degrees. The predominant cell cycle variant in mice cardiomyocytes is binucleation, peaking around postnatal day 7 [45], whereas in humans polyploidization occurs mostly between 10 and 20 years of age [31]. The functional consequences of polyploidization are still unknown. Another interesting question is the relationship between longitudinal and circumferential cardiac growth. Jonker et al. [36] observed that left ventricular myocytes grow more rapidly in width than in length, leading to a significant decline in their length:width ratio. This is typical of concentric hypertrophy and is considered as a feedback response to pressure overload. It is easy to measure myocyte width in cross sections, but far more difficult to obtain the length—it requires myocytes to be oriented strictly parallel with the section plane. It could be performed more easily on isolated myocytes, but then the positional information is lost; it is supposed that there are gradients in size transmurally and between the left and right ventricles. Prenatally, there is mostly hyperplasia, with a bit of hypertrophy in pressure overload [5,46]. This seems to be due to wall strains (circumferential), but there are several mediating cascades, including growth factor signaling [47].

The ontogenetic development of cardiac muscles is accompanied by a corresponding development of the blood vessels and cells, such as fibroblasts, immune cells, neurons, etc. (for Ref. see [48]). Regarding the blood supply, during early prenatal development the heart chambers are spongious and sufficiently thin; therefore, coronary circulation is not necessary. During further development, the spongious myocardium is replaced—as during phylogeny—by a compact layer [21,49]. The first signs of coronary vessels’ formation are the presence of endothelial tubes in the subepicardium; vascularization then proceeds in a predictable sequence. The tubes branch and anastomose, venous vessels are formed and attach to the sinus venosus, and finally the capillary plexus develops [50]. The venous part then connects to the right atrium via the coronary sinus, while the coronary stems penetrate the aortic wall in the left and right aortic sinus slightly later [51]. The further differentiation of the coronary vessel wall is then pressure dependent and involves the addition of the smooth muscle media to the wall of coronary arteries in a baso-apical gradient [50]. The early postnatal period is characterized by a rapid rate of capillarization: nearly half of the adult capillaries in the rat heart are formed within the first 3–4 postnatal weeks [52]. A major quantitative feature of the capillary bed in the subsequent period is the decrease in the fiber-to-capillary ratio and capillary density: the number of cardiac cells supplied by a single capillary decreases from 4 to 6 in the neonatal period to one in the adult hearts of various mammalian species [53]. In adult animals, the number of muscle fibers and capillaries/mm^2^ gradually declines as a result of the growth of the diameter of muscle fibers; the fiber-to-capillary ratio remains constant (1:1), and the diffusion distance becomes longer. In the heart muscles of old animals (in rats over to years of age), the number of muscle fibers/mm^2^ is unchanged, while the number of capillaries/mm^2^ is lower. The result is a significant increase in the fiber-to-capillary ratio and a prolongation of the diffusion distance. The development of the fiber-to-capillary ratio is probably the same in the ontogeny of all mammalian hearts [50]. Normally, the processes of myocardial and coronary development is closely linked [54]; interestingly, the factors controlling the growth of these two compartments are similar, with FGF2 (fibroblast growth factor 2) emerging as a major player [47,55].

## 4. Regulation of Normal Cardiac Growth

What controls the development of cardiac size? How is cardiac growth regulated in order to achieve the target size? These basic questions have received considerable attention, and we are now starting to understand how external and genetic determinants coordinate organ size (for more details, see Refs. [3,4,56]). In mammals, regulatory mechanisms have developed to ensure that the heart reaches the correct size. As mentioned above, cardiac growth during ontogeny is biphasic: hyperplastic growth is replaced by hypertrophic growth shortly after birth. Therefore, interest was first concentrated on the analysis of the cellular mechanisms responsible for the control of cardiomyocyte division. Several recent reports have revealed that proliferation during cardiogenesis is coordinated by growth factors, intrinsic signaling morphogenetic pathways, and cell-cycle regulators [56]. Extrinsic regulatory signals include the fibroblast growth factor (FGF), bone morphogenetic protein, and canonical and non-canonical Wnt signaling pathways (for Ref. see [57]). Sources of such growth factors may be multiple; they are also produced by the myocytes themselves, and released in response to the increased stretch.

**Figure 6:**
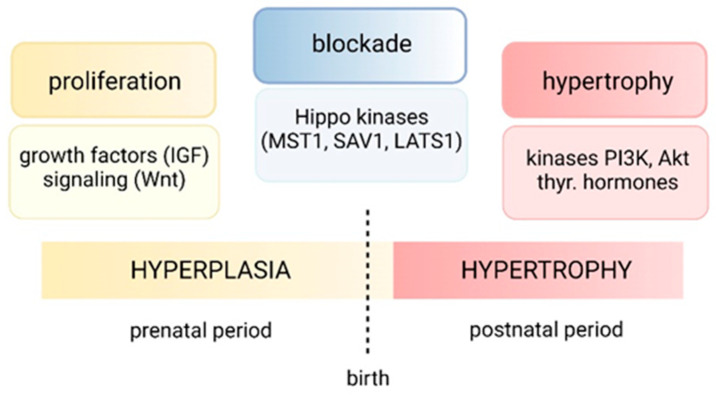
Scheme of regulation of cardiac growth during prenatal and postnatal ontogeny. Main factors responsible for the switch from hyperplastic to hypertrophic growth. Hippo signaling limits proliferation of cardiomyocytes; its role in the regulation in later postnatal growth is still unknown [3].

As mentioned by Sedmera and Thompson [5], “what causes myocyte divide is interesting, but what makes them stop dividing is fascinating”. The cessation of cardiomyocyte proliferation after birth is accompanied by the downregulation of many basic cell-cycle factors and the upregulation of cell-cycle inhibitors [4]. Recently, it has been observed that heart size is intrinsically controlled during development and that the anti-growth Hippo kinase pathway plays a critical role [3] (Figure 6). Hippo limits hyperplastic (rather than hypertrophic) cardiomyocytes’ growth during prenatal and early postnatal development. Whether Hippo signaling also regulates later postnatal cardiac growth is still unknown. Taken together, these studies suggest that, in concert with other signaling pathways, Hippo signaling deregulates heart size by suppressing cardiomyocyte proliferation. Another example is the homeobox protein Meis1; the inactivation of Meis1 in mouse cardiomyocytes extends the postnatal cardiomyocyte proliferation and allows the reactivation of mitosis in the adult heart, whereas the overexpression of Meis1 reduces neonatal proliferation [58].

Shortly after birth, several transitions are triggered in the neonatal heart that initiate a complex remodeling from the fetal state to the adult heart. As noted above, a switch in growth mode occurs in the early postnatal heart, where cardiomyocytes terminally differentiate, cease proliferating, and undergo hypertrophic growth that increases cell diameter and mass, accompanied by a marked increase in heart weight (reaching adult values in mice at approximately 3 months of age [59]). This hypertrophic cardiac growth is regulated by multiple signals such as the phosphoinositide 3 kinase/protein kinase B/insulin (PI3K/AKT/insulin) pathway, thyroid hormones, etc. (e.g., [3]).

**Figure 7:**
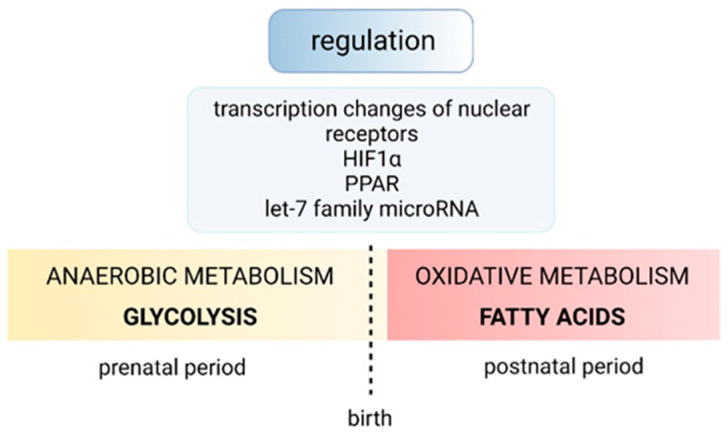
Scheme of regulation of cardiac metabolic switch from a predominantly anaerobic and glycolysis dependent prenatal period to an adult state that uses oxidative metabolism. The switch in metabolism is hypothesized to begin prior to or around the time of birth. It is, however, unknown if a progressive change in energy metabolism instigates the switch from proliferative to quiescent cardiomyocytes, or if the switch from proliferative to quiescent cardiomyocytes drives the change in cardiac energy metabolism [61].

To meet the metabolic demands resulting from the increasing heart size and the workload, the heart undergoes a major metabolic transition in the early postnatal period, from a predominantly anaerobic and glycolysis-dependent fetal-like state to an adult state that utilizes oxidative metabolism (Figure 7). Following this switch, the postnatal heart primarily catabolizes fatty acids, in contrast to the fetal heart, which relies predominantly on carbohydrates as a primary source of energy [60]. The postnatal cardiomyocytes produce a greater amount of ROS, which are generated in part by oxidative phosphorylation. ROS can cause oxidative DNA damage, which has been shown to induce cell-cycle arrest. However, it has been demonstrated that reducing ROS, e.g., by exposing animals to chronic hypoxia, increases cardiomyocyte cell-cycle activity during normal postnatal development (see above [39]).

The metabolic switch from anaerobic to oxidative energy metabolism is regulated by several nuclear receptors and their cofactors, such as the hypoxia-inducible factor (HIF) pathway, the PGC-1/peroxisome proliferator-activated receptor (PPAR) α pathway and the peroxisome proliferator-activated receptor gamma coactivator 1 (PGC-1)/PPAR δ pathway; however, the underlying molecular mechanisms that drive this process have not yet been elucidated [62,63]. The recently revealed role of HIF-1α–mediated hypoxic responses in fetal cardiomyocyte proliferation supports a role of neonatal oxygen exposure in promoting cell-cycle termination. HIF-1 α stabilization is critical for proliferation by reducing genes encoding negative cell-cycle regulators and activating genes favoring the metabolic transition to glycolytic metabolism [64].

## 5. Differences in Cardiac Response to the Increased Workload during Postnatal Ontogeny

Detailed knowledge of the different ontogenetic periods will help predict and explain the adaptive cardiac responses to various pathological stimuli, including increased workload. As early as 1965, Rakusan et al. suggested that the results should be different if the growth stimulus is applied during the early postnatal period, characterized by the combination of hyperplastic and hypertrophic growth, or later, during the exclusively hypertrophic phase of cardiac development [65]. As noted above, the rapid switch from hyperplastic to hypertrophic growth occurs in rats as early as between postnatal days 3 and 4 [29]. Interestingly, postnatal day 4 also represents a critical turning point in the ontogenetic development of the relative heart weight, cardiac contractile performance and inotropic responsiveness [66].

In order to study the effect of increased hemodynamic load on postnatal cardiac development, an experimental model was developed to load the immature heart with additional workload by constricting the abdominal aorta (AC) [67]. This intervention resulted in a much more expressed increase in left ventricular mass than a similar constriction performed in adult animals [68]. Unfortunately, in early studies AC was not applied in rats younger than 5 days of age [69,70,71], likely because of the increased mortality associated with abdominal surgery at younger stages. In our modification [72], the aorta was exposed from the dorsolateral side, causing much less trauma and allowing us to use this model in 2-day-old rats with minimum early mortality. AC at day 2 induced a rapid increase in the absolute and relative heart weight, already apparent at day 3 and reaching statistical significance by day 5, whereas a significant increase in left ventricular cardiomyocyte width first occurred on day 10. The pre-labeling of 3H-thymidine with label dilution showed an increased number of cellular divisions in AC rats, correlating with the severity of the phenotype. The terminal DNA synthesis index increased significantly at day 3, but did not differ from controls at later stages of development. The apoptotic rates were not different from controls at any sampling interval. These results thus suggest that the adaptation of the neonatal heart to increased pressure load is rapid, and is based on transient hyperplasia followed by hypertrophy of ventricular cardiomyocytes [73].

**Figure 8:**
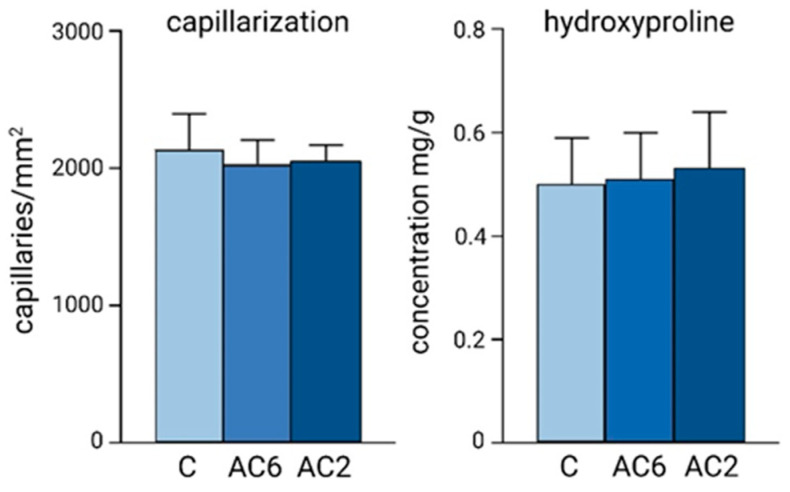
Number of capillaries/mm^2^ and the left ventricular concentration of hydroxyproline (mg/kg dry weight) in the myocardium of control adult rats (C) and in adult rats with gradual pressure overload, induced by abdominal aortic constriction on postnatal day 2 (AC2) or 6 (AC6) (data from [72]).

Pressure overload imposed in rats early after birth resulted in an accelerated biogenesis of the extracellular matrix and capillary angiogenesis [72] (Figure 8). However, unchanged capillary density in both AC groups indicated that angiogenesis was stimulated proportionally to the increase in ventricular mass. Unchanged capillarization is a specific feature of cardiomegaly induced early after birth; on the other hand, hypertrophy caused in adults is generally associated with reduced capillary density [74]. Similarly, the concentration of hydroxyproline in the AC myocardium was unchanged, suggesting that the formation of collagenous proteins was also proportional to the cardiac growth. This means that an increased workload applied early after birth leads to cardiomegaly without myocardial fibrosis. This situation is principally different from the increased workload induced in adults, which results in the increased accumulation of collagen and myocardial fibrosis [75,76].

Recently, Mohammadi et al. have precisely described the neonatal mouse model of pressure overload induced by transverse AC applied at postnatal days 1 and 7 [77,78]. Surgery performed on day 7 induced cardiac dysfunction, fibrosis and the hypertrophy of cardiomyocytes, similar to results in adult animals. In contrast, AC induced in 1-day-old animals largely prevented these maladaptive changes and was associated with increased angiogenesis and cardiomyocyte proliferation.

In conclusion, an increased workload induced in animals soon after birth (i.e., before the switch from hyperplastic to hypertrophic growth) leads to a specific type of left ventricular enlargement which, in contrast to the same stimulus applied in adulthood, is characteristic by hyperplasia of the cardiomyocytes, capillary angiogenesis and biogenesis of collagenous proteins proportional to the growth of myocytes. Although neonatal rats may differ in maturity from neonatal humans, these studies suggest that timing may be of crucial importance in neonatal cardiac interventions. They support the current practice of early definitive repairs to selected congenital cardiac anomalies: such an approach can result in the remodeling of myocardial architecture more through hyperplasia rather than hypertrophy, which may be more beneficial for long-term results of surgical treatment in pediatric cardiology [73]. Unfortunately, the molecular mechanisms responsible for the developmental differences in cardiac responses to increased workload are still unknown. More developmental studies are, therefore, necessary to expand our knowledge in this respect.

## 6. Concluding Remarks

This review summarizes the recent data on cardiac adaptation to increased workload during phylogenetic and ontogenetic development, with particular attention to the transition from hyperplastic to hypertrophic growth. It supports the view that developmental biology and medicine form a rational combination of individual biomedical science, describing the functional adaptation of a particular system, and evolution, which is the science of how biological systems came to exist.

The importance of the developmental approach for experimental and clinical cardiology is indisputable. Clinical–epidemiological studies have clearly demonstrated that risk factors of serious cardiovascular diseases are already present in the early stages of ontogenetic development, and genetic factors are present even before birth. Experimental studies on the pathogenetic mechanisms of these disturbances must therefore move to the early developmental periods. The investigation of developmental aspects of cardiac adaptation to increased workload may serve as a good example. Moreover, congenital cardiac malformations are still the most common cause of infant mortality from congenital defects in developed countries. It is, therefore, not surprising that the interests of both theoretical and clinical cardiologists in the developmental approach continues to grow. Recent progress in molecular cardiology and the promising possibilities of human prenatal cardiology [79] have significantly accelerated this trend.

## References

[1] Adolph E.F. (1956). General and specific characteristics of physiological adaptations. Am. J. Physiol..

[2] Ostadal B., Ostadal B., Dhalla N.S. (2014). Comparative aspects of cardiac adaptation. Cardiac Adaptations.

[3] Heallen T.R., Kadow Z.A., Wang J., Martin J.F. (2020). Determinants of Cardiac Growth and Size. Cold Spring Harb. Perspect. Biol..

[4] Yuan X., Braun T. (2017). Multimodal Regulation of Cardiac Myocyte Proliferation. Circ. Res..

[5] Sedmera D., Thompson R.P. (2011). Myocyte proliferation in the developing heart. Dev. Dyn. Off. Publ. Am. Assoc. Anat..

[6] Zhu F., Meng Q., Yu Y., Shao L., Shen Z. (2021). Adult Cardiomyocyte Proliferation: A New Insight for Myocardial Infarction Therapy. J. Cardiovasc. Transl. Res..

[7] Hesse R. (1921). Das Herzgewicht der Wirbeltiere. Zool. Jahrb. Abt. Allg. Zool. Physiol..

[8] Poupa O., Ostadal B. (1969). Experimental cardiomegalies and “cardiomegalies” in free-living animals. Ann. N. Y. Acad. Sci..

[9] Clark A.J. (1927). Comparative Physiology of the Heart.

[10] Poupa O., Rakusan K., Ostadal B. (1970). The effect of physical activity upon the heart of vertebrates. Medicine and Sport Science.

[11] Poupa O., Ostadal B., Dhalla N.S. (1993). Heart story: A view to the past. Heart Function in Health and Disease.

[12] Farrell A.P., Smith F., Gamprl A.K., Gillis T.E., Farrell A.P., Brauner C.J. (2017). Cardiac form, function and physiology. The Cardiovascular Systém: Morphology, Control and Function. Fish Physiology.

[13] Jones D.R., Brill R.W., Bushnell P.G. (1993). Ventricular and Arterial Dynamics of Anaesthetised and Swimming Tuna. J. Exp. Biol..

[14] Icardo J.M., Gamprl A.K., Gillis T.E., Farrell A.P., Brauner C.J. (2017). Heart morphology and anatomy. The Cardiovascular Systém: Morphology, Control and Function. Fish Physiology.

[15] Ostadal B., Ostadalova I., Dhalla N.S. (1999). Development of cardiac sensitivity to oxygen deficiency: Comparative and ontogenetic aspects. Physiol. Rev..

[16] Ostadal B., Schiebler T.H. (1971). The terminal blood bed in the heart of fish. Z. Fur Anat. Entwickl..

[17] Ostadal B., Schiebler T.H., Rychter Z. (1975). Relations between development of the capillary wall and myoarchitecture of the rat heart. Adv. Exp. Med. Biol..

[18] Bass A., Ostadal B., Pelouch V., Vitek V. (1973). Differences in weight parameters, myosin-ATPase activity and the enzyme pattern of energy supplying metabolism between the compact and spongious cardiac musculature of carp (*Cyprinus carpio*) and turtle (*Testudo horsfieldi*). Pflug. Arch. Eur. J. Physiol..

[19] Tota B., Garofalo F., Sedmera D., Wang T. (2012). Fish heart growth and function: From gross morphology to cell signaling and back. Cardiac nonuniformity: From genes to shape. Ontogeny and Phylogeny of the Vertebrate Heart.

[20] Farrell A.P., Farrell N.D., Jourdan H., Cox G., Sedmera D., Wang T. (2012). A perspective on the evolution of the coronary circulation in fishes and the transition to terrestrial life. Ontogeny and Phylogeny of the Vertebrate Heart.

[21] Ostadal B., Rychter Z., Poupa O. (1970). Comparative aspects of the development of the terminal vascular bed in the myocardium. Physiol. Bohemoslov..

[22] Becker R.O., Chapin S., Sherry R. (1974). Regeneration of the ventricular myocardium in amphibians. Nature.

[23] Chablais F., Veit J., Rainer G., Jaźwińska A. (2011). The zebrafish heart regenerates after cryoinjury-induced myocardial infarction. BMC Dev. Biol..

[24] Zuppo D.A., Tsang M. (2020). Zebrafish heart regeneration: Factors that stimulate cardiomyocyte proliferation. Semin. Cell Dev. Biol..

[25] Burggren W.W., Warburton S.J. (1994). Patterns of form and function in developing hearts: Contributions from non-mammalian vertebrates. Cardioscience.

[26] Burggren W.W. (1988). Cardiac design in lower vertebrates: What can phylogeny reveal about ontogeny?. Experientia.

[27] Birkedal R., Laasmaa M., Branovets J., Vendelin M. (2022). Ontogeny of cardiomyocytes: Ultrastructure optimization to meet the demand for tight communication in excitation-contraction coupling and energy transfer. Philos. Trans. R. Soc. Lond. Ser. B Biol. Sci..

[28] Rumyantsev P.P. (1982). Cardiomyocytes in the Processes of Reproduction, Differentiation, and Regeneration.

[29] Li F., Wang X., Capasso J.M., Gerdes A.M. (1996). Rapid transition of cardiac myocytes from hyperplasia to hypertrophy during postnatal development. J. Mol. Cell. Cardiol..

[30] Porrello E.R., Mahmoud A.I., Simpson E., Hill J.A., Richardson J.A., Olson E.N., Sadek H.A. (2011). Transient regenerative potential of the neonatal mouse heart. Science.

[31] Bergmann O., Zdunek S., Felker A., Salehpour M., Alkass K., Bernard S., Sjostrom S.L., Szewczykowska M., Jackowska T., Dos Remedios C. (2015). Dynamics of Cell Generation and Turnover in the Human Heart. Cell.

[32] Mollova M., Bersell K., Walsh S., Savla J., Das L.T., Park S.Y., Silberstein L.E., Dos Remedios C.G., Graham D., Colan S. (2013). Cardiomyocyte proliferation contributes to heart growth in young humans. Proc. Natl. Acad. Sci. USA.

[33] Rychter Z., Rychterova V., Lemez L. (1979). Formation of the heart loop and proliferation structure of its wall as a base for ventricular septation. Herz.

[34] Kirby M.L., Gale T.F., Stewart D.E. (1983). Neural crest cells contribute to normal aorticopulmonary septation. Science.

[35] Kajstura J., Mansukhani M., Cheng W., Reiss K., Krajewski S., Reed J.C., Quaini F., Sonnenblick E.H., Anversa P. (1995). Programmed cell death and expression of the protooncogene bcl-2 in myocytes during postnatal maturation of the heart. Exp. Cell Res..

[36] Jonker S.S., Louey S., Giraud G.D., Thornburg K.L., Faber J.J. (2015). Timing of cardiomyocyte growth, maturation, and attrition in perinatal sheep. FASEB J. Off. Publ. Fed. Am. Soc. Exp. Biol..

[37] Anversa P., Cheng W., Liu Y., Leri A., Redaelli G., Kajstura J. (1998). Apoptosis and myocardial infarction. Basic Res. Cardiol..

[38] Johnson J., Mohsin S., Houser S.R. (2021). Cardiomyocyte Proliferation as a Source of New Myocyte Development in the Adult Heart. Int. J. Mol. Sci..

[39] Nakada Y., Canseco D.C., Thet S., Abdisalaam S., Asaithamby A., Santos C.X., Shah A.M., Zhang H., Faber J.E., Kinter M.T. (2017). Hypoxia induces heart regeneration in adult mice. Nature.

[40] Faeh D., Moser A., Panczak R., Bopp M., Röösli M., Spoerri A. (2016). Independent at heart: Persistent association of altitude with ischaemic heart disease mortality after consideration of climate, topography and built environment. J. Epidemiol. Community Health.

[41] Johnson J., Yang Y., Bian Z., Schena G., Li Y., Zhang X., Eaton D.M., Gross P., Angheloiu A., Shaik A. (2023). Systemic Hypoxemia Induces Cardiomyocyte Hypertrophy and Right Ventricular Specific Induction of Proliferation. Circ. Res..

[42] Leone M., Magadum A., Engel F.B. (2015). Cardiomyocyte proliferation in cardiac development and regeneration: A guide to methodologies and interpretations. Am. J. Physiol. Heart Circ. Physiol..

[43] Auchampach J., Han L., Huang G.N., Kühn B., Lough J.W., O’Meara C.C., Payumo A.Y., Rosenthal N.A., Sucov H.M., Yutzey K.E. (2022). Measuring cardiomyocyte cell-cycle activity and proliferation in the age of heart regeneration. Am. J. Physiol. Heart Circ. Physiol..

[44] Zebrowski D.C., Engel F.B. (2013). The cardiomyocyte cell cycle in hypertrophy, tissue homeostasis, and regeneration. Rev. Physiol. Biochem. Pharmacol..

[45] Alkass K., Panula J., Westman M., Wu T.D., Guerquin-Kern J.L., Bergmann O. (2015). No Evidence for Cardiomyocyte Number Expansion in Preadolescent Mice. Cell.

[46] Pesevski Z., Sedmera D., Ostadal B., Dhalla N.S. (2013). Prenatal adaptations to overload. Cardiac Adaptations.

[47] Krejci E., Pesevski Z., Nanka O., Sedmera D. (2016). Physiological role of FGF signaling in growth and remodeling of developing cardiovascular system. Physiol. Res..

[48] Laflamme M.A., Murry C.E. (2011). Heart regeneration. Nature.

[49] Sedmera D., Pexieder T., Hu N., Clark E.B. (1997). Developmental changes in the myocardial architecture of the chick. Anat. Rec..

[50] Tomanek R.J. (2016). Developmental Progression of the Coronary Vasculature in Human Embryos and Fetuses. Anat. Rec..

[51] Velkey J.M., Bernanke D.H. (2001). Apoptosis during coronary artery orifice development in the chick embryo. Anat. Rec..

[52] Rakusan K., Turek Z. (1985). Protamine inhibits capillary formation in growing rat hearts. Circ. Res..

[53] Rakusan K., Zak R. (1984). Cardiac growth, maturation and ageing. Growth of the Heart in Health and Disease.

[54] Lavine J.S., Poss M., Grenfell B.T. (2008). Directly transmitted viral diseases: Modeling the dynamics of transmission. Trends Microbiol..

[55] Tomanek R.J., Ishii Y., Holifield J.S., Sjogren C.L., Hansen H.K., Mikawa T. (2006). VEGF family members regulate myocardial tubulogenesis and coronary artery formation in the embryo. Circ. Res..

[56] Heallen T.R., Kadow Z.A., Kim J.H., Wang J., Martin J.F. (2019). Stimulating Cardiogenesis as a Treatment for Heart Failure. Circ. Res..

[57] Galdos F.X., Guo Y., Paige S.L., VanDusen N.J., Wu S.M., Pu W.T. (2017). Cardiac Regeneration: Lessons from Development. Circ. Res..

[58] Mahmoud A.I., Kocabas F., Muralidhar S.A., Kimura W., Koura A.S., Thet S., Porrello E.R., Sadek H.A. (2013). Meis1 regulates postnatal cardiomyocyte cell cycle arrest. Nature.

[59] Leu M., Ehler E., Perriard J.C. (2001). Characterisation of postnatal growth of the murine heart. Anat. Embryol..

[60] Lopaschuk G.D., Collins-Nakai R.L., Itoi T. (1992). Developmental changes in energy substrate use by the heart. Cardiovasc. Res..

[61] Dimasi C.G., Darby J.R.T., Morrison J.L. (2023). A change of heart: Understanding the mechanisms regulating cardiac proliferation and metabolism before and after birth. J. Physiol..

[62] Chung S., Dzeja P.P., Faustino R.S., Perez-Terzic C., Behfar A., Terzic A. (2007). Mitochondrial oxidative metabolism is required for the cardiac differentiation of stem cells. Nat. Clin. Pract. Cardiovasc. Med..

[63] Lopaschuk G.D., Jaswal J.S. (2010). Energy metabolic phenotype of the cardiomyocyte during development, differentiation, and postnatal maturation. J. Cardiovasc. Pharmacol..

[64] Guimarães-Camboa N., Stowe J., Aneas I., Sakabe N., Cattaneo P., Henderson L., Kilberg M.S., Johnson R.S., Chen J., McCulloch A.D. (2015). HIF1α Represses Cell Stress Pathways to Allow Proliferation of Hypoxic Fetal Cardiomyocytes. Dev. Cell.

[65] Rakusan K., Jelinek J., Korecky B., Soukupova M., Poupa O. (1965). Postnatal development of muscle fibers and capillaries in the rat heart. Physiol. Bohemoslov..

[66] Ostadalova I., Kolar F., Ostadal B., Rohlicek V., Rohlicek J., Prochazka J. (1993). Early postnatal development of contractile performance and responsiveness to Ca^2+^, verapamil and ryanodine in the isolated rat heart. J. Mol. Cell. Cardiol..

[67] Dowell R.T., McManus R.E. (1978). Pressure-induced cardiac enlargement in neonatal and adult rats. Left ventricular functional characteristics and evidence of cardiac muscle cell proliferation in the neonate. Circ. Res..

[68] Rakusan K., Korecky B. (1985). Regression of cardiomegaly induced in newborn rats. Can. J. Cardiol..

[69] Campbell S.E., Rakusan K., Gerdes A.M. (1989). Change in cardiac myocyte size distribution in aortic-constricted neonatal rats. Basic Res. Cardiol..

[70] Campbell S.E., Korecky B., Rakusan K. (1991). Remodeling of myocyte dimensions in hypertrophic and atrophic rat hearts. Circ. Res..

[71] Yamamoto H., Avkiran M. (1993). Left ventricular pressure overload during postnatal development. Effects on coronary vasodilator reserve and tolerance to hypothermic global ischemia. J. Thorac. Cardiovasc. Surg..

[72] Kolar F., Papousek F., Pelouch V., Ostadal B., Rakusan K. (1998). Pressure overload induced in newborn rats: Effects on left ventricular growth, morphology, and function. Pediatr. Res..

[73] Sedmera D., Thompson R.P., Kolar F. (2003). Effect of increased pressure loading on heart growth in neonatal rats. J. Mol. Cell. Cardiol..

[74] Rakusan K., Legato M.J. (1987). Microcirculation in the stressed heart. The Stressed Heart.

[75] Weber K.T., Brilla C.G., Janicki J.S. (1993). Myocardial fibrosis: Functional significance and regulatory factors. Cardiovasc. Res..

[76] Czubryt M.P., Hale T.M. (2021). Cardiac fibrosis: Pathobiology and therapeutic targets. Cell. Signal..

[77] Malek Mohammadi M., Abouissa A., Azizah I., Xie Y., Cordero J., Shirvani A., Gigina A., Engelhardt M., Trogisch F.A., Geffers R. (2019). Induction of cardiomyocyte proliferation and angiogenesis protects neonatal mice from pressure overload-associated maladaptation. JCI Insight.

[78] Malek Mohammadi M., Abouissa A., Heineke J. (2021). A surgical mouse model of neonatal pressure overload by transverse aortic constriction. Nat. Protoc..

[79] Hunter L.E., Seale A.N. (2018). EDUCATIONAL SERIES IN CONGENITAL HEART DISEASE: Prenatal diagnosis of congenital heart disease. Echo Res. Pract..

